# Supplementary material for: Third-party evaluators perceive AI as more compassionate than expert humans
Source: Commun Psychol. 2025 Jan 10;3:4. doi: 10.1038/s44271-024-00182-6 (PMC11723910; doi:10.1038/s44271-024-00182-6)
Supplement: Supplementary file 2 — Supplementary Information [file 44271_2024_182_MOESM2_ESM.pdf]

Supporting Information for:

Third-Party Evaluators Perceive AI as More Compassionate than Expert Humans

Dariya Ovsyannikova[a] (dariya.ovsyannikova@mail.utoronto.ca)

Victoria Oldemburgo de Mello[a,c] (victoria.mello@mail.utoronto.ca)

Michael Inzlicht[a,b,c*] (michael.inzlicht@utoronto.ca)

[a] University of Toronto, Department of Psychology, Canada

[b] Rotman School of Management, University of Toronto, Canada

[c] These authors jointly supervised this work.

***Corresponding author:** michael.inzlicht@utoronto.ca

This file includes:

Supplementary Methods

Supplementary notes (S1), (S2), and (S3)

Supplementary Figures 1-6

Supplementary references (1-6)

Other supporting materials for this manuscript include the following:

Data, Code, and Materials Availability for Studies 1-4 (https://osf.io/wjx48/)

Dataset S1 and S2 (https://osf.io/wjx48/)

R code S1 and S2 (https://osf.io/wjx48/)

Survey S1 and S2 (https://osf.io/wjx48/)

Supplementary Methods

1. Detailed measures and procedures

Data collection was conducted in two stages: prompt generation/validation and response generation. Human responses were elicited via online questionnaires administered through the SONA systems and Prolific Academic platforms, while AI responses were generated using ChatGPT, mirroring the prompt conditions given to human participants. Details of the measurements used in our analyses are thoroughly documented in the main manuscript, but here, we provide greater detail on the perceived responsiveness[1] measure and all other exploratory measurement(s) we did not include in our main manuscript. The full dataset for our supplementary study (S1) and (S2) (the findings for which we report in Supplementary Note 1 and Supplementary Note 2, respectively) is available at https://osf.io/wjx48/. Similarly, the survey for supplementary study 1 (S1) and 2 (S2), as well as the prompt generation surveys used for all the studies can be found at https://osf.io/wjx48/.

Prompt generation and validation

Initially, we generated 18 sample prompts/vignettes for the four iterations of our study; these vignettes described positive–individuals' feelings of joy of gratitude–and negative–individuals' feelings of suffering–life circumstances and were inspired by life events that the general public could find relatable (e.g., the death of a close person, a major life transition, admission into university, etc.).

These vignettes were then presented to and validated by 8 fellow lab members in rank order fashion. The lab members completed an online questionnaire in which they were presented with 10 negative and 8 positive vignettes and were asked to rank the choices from 1-10 in terms of content quality, details, and emotional salience, keeping in mind their top 5 negative and positive prompts with respect to the above ranking criteria. After each set of prompts (positive and negative), they were asked to provide a short rationale regarding their top 5 picks from each valence category. Following completion of the questionnaire, we obtained the mode of the rank order for each of the two valence categories to finalize our top 5 positive and negative vignettes. All the selected vignettes and associated human responses can be found under the *Materials* folder at https://osf.io/wjx48/. At present, see sample scenarios below:

Negative vignette:

"Recently, I have been struggling with confusion over my current social role. I feel depressed. I had just retired a few weeks ago and now I feel like I have no purpose, no people around me to turn to. I don't know who to turn to or what new activities I can start, and getting up every day has become more and more psychologically difficult".

Positive Vignette:

"I went from feeling depressed and isolated to forcing myself to attend the gym and building healthy life habits. 6 months later, I am now training to run a marathon in my city! Finding the motivation to train can be difficult, but the high that you feel from running and supporting those alongside you, those who have fought to be in the same place, is unmatched".

Human response generation

We piloted our selected 10 vignettes in an online questionnaire given to 10 first-year psychology students at the University of Toronto Scarborough (UTSC) through the SONA systems interface[2] to assess the individuals' general response tendencies (i.e. did they adhere to the instructions; how much did they write when prompted with a suggested word count). Furthermore, we were informally interested in assessing participants' response pattern–whether response length, detail, and level of compassion differed based on vignette valence (whether the vignette depicted a negative life circumstance, like the death of a loved one, or a positive life circumstance, like acceptance into university) and the progression of time (i.e. were fatigue effects observed with respect to response quality as a function of time progression). Each participant was asked to be supportive towards the hypothetical individuals across 10 vignettes, providing compassionate responses but straying away from providing any advice.

Following the SONA pilot, we provided the same questionnaire to 10 participants from Canada and the US on Prolific Academic[3] to obtain human responses that were more representative of the general population and to generate a mean word count that we could use to standardize the response instructions for our sample AI responders. The response instructions for both human and AI response authors are displayed in Supplementary Figure 1, Section 2 of the Methods.

As with the vignettes, we had 7 members of our lab (3 graduate students and 4 research assistants who were not involved in the study and were blind to its purpose) rank order the questionnaire responses; members were asked to rank their top 5 participants and evaluate their responses' strength based on response consistency, reflecting high quality of content, details, emotional salience, and relatability to the circumstances described in each vignette. Finally, we obtained the participant rank order mode to select the top 5 individuals that would constitute the human responder sample for our following comparison study iterations; this provided us with 50 data points, or 10 data points per participant.

Artificial Intelligence (ChatGPT) response generation

To generate a set of AI responses for the following comparison studies, we presented the same 10 validated vignettes (pos/neg) and response instructions to ChatGPT across 5 different chats–each chat functioned as a separate AI responder, allowing for differences in response styles and content. Responses from these chats were then used towards our first prolific study (see main manuscript) comparing differences in compassion evaluations for AI and human responders, as well as subsequent study iterations. All the selected vignettes and associated AI responses can be found in survey-format under the *Materials* folder at https://osf.io/wjx48/.

Measures

Compassion and Response Preference. Both the compassion items and binary forced choice question were inspired by the methods used in a recent study[4] in which researchers examined third-party evaluations of empathy for AI and physician-generated responses in a reddit forum, using a binary forced choice measure to assess which response participants thought was better, and using items pertaining to empathy that were rated on a 5-point likert scale.

Responsiveness. Perceived responsiveness was measured by having participants assess each response (AI or human-authored) for how well it understood, validated, and cared for the circumstances of the hypothetical individual in each prompt, with three items measuring each responsiveness facet. The three items for the "Understanding", "Validation", and "Caring" facets were selected from the *Microanalytic responsive behaviors coding guide* in the Appendix section of the paper by Maisel et al.[1], based on how contextually appropriate the items were for our study; we further reworded some of the items to better apply to the context of the authored responses. All responses were recorded in a 5-point Likert scale ranging from 1 (Strongly Disagree) to 5 (Strongly Agree) and averaged per empathic response to create a composite responsiveness score and three separate means for each facet. The survey items for the responsiveness measure, as well as the items for the measures of compassion, response preference, and rating instructions for each transparency condition (used for studies 1-4 in the main manuscript) are available in Supplementary Figures 2-6, in Section 2 of the Methods.

Across our experiments, we also collected data for and measured (but did not analyze) participants' trait level empathy using the Interpersonal Reactivity Index[5].

Empathy. Participants' trait level empathy was measured across 4 categories of the Interpersonal Reactivity Index (IRI), a validated self-report scale that has been utilized in experimental studies to examine empathy in relation to social skills, interpersonal relationships, and various social, personality, and behavioral variables[5]. The IRI is a 28-item questionnaire that seeks to measure individual differences in empathy across 4, 7-item subscales: the Fantasy Scale, Perspective Taking Scale, Empathic Concern Scale, and Personal Distress Scale, with around two reverse-coded items in each subscale[5].

In a review of the Interpersonal Reactivity Scale's reliability and validity, a moderate convergent validity of the scale with other empathy measures ($.11 < r < .63$) and adequate construct validity in accordance with Confirmatory Factor Analysis (CFA) model fit parameters

(CFI: .956 to .962) was noted[5]. In the present study, participants were asked to indicate how well each of the 28 items described them on a 5-point Likert scale, with responses ranging from 0 (Does not describe me well) to 4 (Describes me very well).

Sampling Strategy

In studies 1 and 4 (as well as the exploratory analysis we report in Supplementary Note 1), which used a fully within-subjects framework, our target was 54 participants based on power analysis predictions that this size would provide at least 80% probability of detecting a typical social psychology effect size of d = .4[6]. For studies 2 and 3, employing a mixed-methods approach with one between-subjects and one within-subjects factor, our goal was to include 400 participants. This sample size was determined to achieve 80% power to identify an effect as minor as f = 0.15, accounting for potential dropout due to participant inattention. Together, the sampling strategies were used to yield N = 54 and N = 58 participants for studies 1 and 4, respectively, as well as N = 197 and N = 247 participants for studies 2 and 3, respectively.

Preregistration

We preregistered our study protocol for studies 1-4 at aspredicted.org; please find the preregistration links for study 1 (#145328; https://aspredicted.org/F8L_JSZ), study 2 (#153555; https://aspredicted.org/QWQ_FNM), study 3 (#161352; https://aspredicted.org/M9G_VKK), and study 4 (#173893; https://aspredicted.org/YTQ_2M9). We would like to disclose that, for our study 1 preregistration, we revised our analysis for response preferences from a dependent means t-test to a one sample t-test due to a typo. Furthermore, we prematurely collected data prior to submitting our preregistered protocol for study 2. However, while we had already collected data two days prior, we did not look at the data (i.e., we did not download or assess any data files), nor compensated participants who completed the survey (to avoid viewing the data provided by participants). Participants were made aware of the delay in compensation due to manual review following the study's completion in the debriefing section of the survey. We wish to disclose this information to justify Study 2 as preregistered and to be transparent.

2. Instructions and Questionnaire Items Given to Participants.

In this section, we present the questionnaire items that were used to instruct human and AI authors during the response generation phase of the study, as well as the items that were used to measure third-party evaluators' compassion ratings, preferences, and responsiveness ratings for human and AI-authored responses across studies 1-4 in our main manuscript. These items were also used in our exploratory study, the findings for which are discussed in Supplementary Note 1.

Supplementary Fig. 1: Prompt Generation Instructions for AI (ChatGPT-4) and Human Authors.

Qiv

In the next part of the study, we will give you a series of prompts that detail the experiences of individuals with different life circumstances. Some of these prompts/statements describe negative life circumstances in which the individuals feel suffering, whereas others describe positive circumstances in which individuals feel joy or gratitude. After reading these statements, we ask that you provide a written response to these individuals. Specifically, we want you to be compassionate when you respond, but avoid giving advice. You are not limited on the amount of content that you write and, on average, past participants have written a **minimum of 50-75 word responses**. Furthermore, we are not evaluating your responses based on your writing quality nor your experience with providing social support. We only encourage you to write freely.

The prompt generation instructions were provided to both human Prolific (select and expert) and AI (ChatGPT-4) response authors in a Qualtrics questionnaire that followed the initial SONA pilot.

Supplementary Fig. 2: Blind Condition Instructions for Third-Party Raters.

Qiv

In the next part of the study, we will give you a series of scenarios that detail the experiences of individuals with different life circumstances. Some of these prompts/scenarios describe negative life circumstances in which the individuals feel suffering, whereas others describe positive circumstances in which individuals feel joy or gratitude. Following each statement are two responses from two different individuals; these responses seek to relay compassion to the individual in the above scenario. After reading each response, we ask that you evaluate the **emotional quality** of each response by providing your agreement with a series of statements describing the response on a 5-point rating scale (1 being *Strongly disagree*, and 5 being *Strongly agree*).

The instructions were provided to third-party Prolific evaluators across studies 1-3, under both fixed-blind and manipulated transparency conditions. Under manipulated transparency, only participants assigned to the blind condition would see these instructions, which presented the human and AI (ChatGPT-4) response authors as “two different individuals”.

Supplementary Fig. 3: Transparent Condition Instructions for Third-Party Raters.

> In the next part of the study, we will give you a series of scenarios that detail the experiences of individuals with different life circumstances. Some of these prompts/scenarios describe negative life circumstances in which the individuals feel suffering, whereas others describe positive circumstances in which individuals feel joy or gratitude. Following each statement are two responses from two different agents- **experts who specialize in providing support services; one of these agents is human and one is AI (Artificial Intelligence)**. These responses will be presented in random order and seek to relay compassion to the individual in the above scenario. After reading each response, we ask that you evaluate the **emotional quality** of each response by providing your agreement with a series of statements describing the response on a 5-point rating scale (1 being *Strongly disagree*, and 5 being *Strongly agree*).

The instructions were provided to third-party Prolific evaluators across studies 2-4, under both fixed-transparent and manipulated transparency conditions. Under manipulated transparency, only participants assigned to the transparent condition would see these instructions and associated response author labels (“AI response”, “Human response”).

Supplementary Fig. 4: Questionnaire Items for the Compassion Measure.

Please evaluate **Response A** by indicating the extent to which you agree or disagree with the statements regarding the aforementioned response using the 5-point scale below. Specifically, the 1 on the scale reflects strong disagreement with the statement(s), while 5 reflects strong agreement.

	1- Strongly disagree	2- Disagree	3- Neither agree nor disagree	4- Agree	5- Strongly agree
The response accurately reflects the emotional state of the individual involved	○	○	○	○	○
The response was compassionate	○	○	○	○	○
The response feels impersonal and lacks emotional connection	○	○	○	○	○

The compassion items were presented to third party evaluators following the presentation of each AI and human-authored prompt, across 4 studies (Main Text). Depending on the experimental condition (blind, transparent) to which participants were assigned, the labels for each response author were either generically (Response A, B) or explicitly (AI, Human Response) labeled. The last compassion item/statement is reverse coded.

Supplementary Fig. 5: Questionnaire Item for the Response Preference Measure.

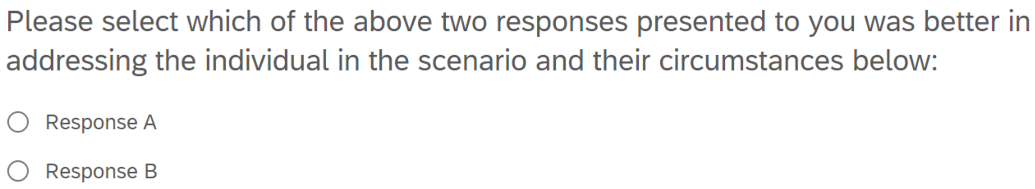

Please select which of the above two responses presented to you was better in addressing the individual in the scenario and their circumstances below:

- ○ Response A
- ○ Response B

The binary forced-choice items were presented to third party evaluators across 4 studies following the compassion items. Depending on the experimental condition (blind, transparent) to which participants were assigned, the labels for each response author were either generically (Response A, B) or explicitly (AI, Human Response) labeled. Each item was dummy coded, where 0 was used to denote a human response, and 1 an AI response.

Supplementary Fig. 6: Questionnaire Items for the Responsiveness Measure in Study 4.

	1- Strongly disagree	2- Disagree	3- Neither agree nor disagree	4- Agree	5- Strongly agree
The response accurately reflects the emotional state of the individual involved	○	○	○	○	○
The response was compassionate	○	○	○	○	○
The response feels impersonal and lacks emotional connection	○	○	○	○	○
The response summarizes the individual's scenario/circumstances and/or repeats back key phrases to the individual.	○	○	○	○	○
The response asks questions about the individual's story, as well as for more details (e.g., "What happened after that?", "Have you thought about getting help with this?").	○	○	○	○	○
The response voices understanding (e.g., "I understand", "I see").	○	○	○	○	○
The response expresses agreement with the individual or takes their side by telling the individual that they were right or agreeing with the cause of the event and/or circumstances (e.g., It wasn't your fault).	○	○	○	○	○
The response describes and/or acknowledges the individual's feelings and emotions, validating that their emotions are justified (e.g., "That must make you really happy/angry").	○	○	○	○	○
The response uses exclamations or judgments: "That's great!"; "That's awful"; or offers congratulations; "Wow!"	○	○	○	○	○
The response expresses empathy or emotions towards the individual in the scenario (e.g., "I'm so happy for you"; "I'm sorry that happened"; "That makes me angry too").	○	○	○	○	○
The response offers support or concern or comfort to the individual in the scenario (e.g., "I'll always be here for you"; "I'm here if you need to talk").	○	○	○	○	○
The response emphasizes that the responder shares in the outcomes of the individual's scenario and/or circumstances (e.g., expressing, "We'll get through this together").	○	○	○	○	○

The responsiveness items were integrated with the three compassion items (items 1-3) and presented following the response from either response author. Compassion item 3 is reverse coded.

Supplementary Note 1

This supplementary study aimed at assessing whether the pattern of preferences for AI-generated responses would hold when responses from both human and AI responders were of equivalent word length. Word length was standardized to match the number of words for each of the 10 human responses.

The results of this study were not included in the main manuscript on account of the human vs. human-edited AI comparison raising concerns about content validity. Manually editing AI responses constrains its original responses, potentially skewing the comparison and inaccurately depicting the behavior of AI systems.

The moderating effect of equivalent word length on ratings for AI and human responses

This study was a replication of study 1, but the AI responses were edited to match the length of the human responses. In addition, all responses were blind, so participants could not see the response author labels. We found both a main effect for response author, $F(1, 312) = 5.16$, $p = 0.02$, and an interaction effect between author and valence, $F(1, 312) = 29.34$, $p < .001$, such that the AI responses ($M = 3.71$, $SD = .61$) had more advantage over the human responses ($M = 3.58$, $SD = .63$) in the negative scenarios, $B = 0.07$, $SE = .03$, $p = 0.01$, than in the positive scenarios, $B = -0.18$, $SE = .05$ $p < .001$. In fact, in the positive scenarios, the human responses ($M = 3.88$, $SD = .56$) were rated as slightly more compassionate than the AI responses ($M = 3.50$, $SD = .71$), suggesting, again, that the AI responses perform better when addressing negative prompts.

Finally, we tested response preference against chance. Unlike the previous experiments, however, we found that participants did not prefer any of the responses above chance level, $t(62) = .36$, $p = .72$, $d = .05$, 95% CI = [-0.20, 0.29], suggesting that response length could have accounted for response preferences in experiments 1-4 (see main manuscript).

Supplementary Note 2

This supplementary study aimed to clarify whether human expert (crisis responder) responses were evaluated as significantly more compassionate and preferred to responses generated by human non-experts. This was done to assess whether the assumption that the expert responses would outperform the non-expert responses in empathic quality, which would add additional nuance to the methodology in the main manuscript and to the interpretation of the findings from study 3 (main manuscript), where AI responses were rated as expressing greater compassion and were preferred over human expert responses.

The results of this study were not included in the main manuscript as this was a tangential question, exploring whether experts are rated as providing more compassionate responses than a select sample of non-experts. Though crisis line workers receive extensive training, their job entails more than generating empathy. Contrastingly, our non-expert sample was specifically selected for being high in expressed empathy. Thus, it is an open question whether experts will be preferred over select, non-experts. As this was an exploratory study, it was not preregistered.

Comparing compassion and preference ratings for blinded human expert and non-expert responses

This study replicated the structure of study 1(main manuscript). Third-party participants ($n = 55$) were exposed to human expert and select non-expert responses to four common vignettes; no AI responses were included in the present study. All responses were blind, so participants could not see the response author labels. We found that the rated compassion for human expert ($M = 3.67$, $SD = .55$) and select non-expert responses ($M = 3.81$, $SD = .58$) were evaluated as being no different, $t(54) = 1.59$, $p = .118$, $d = .21$. We also tested response preference against no preference (0.5) and again found no significant difference in participants' preference for human expert or select non-expert responses, $t(54) = 1.22$, $p = 0.229$, $d = 0.16$, 95% CI = [-0.10, 0.43]. Taken together, these findings suggest that the expert and select non-expert responses did not differ in their compassionate quality or preference.

Supplementary Note 3

This supplementary data section displays our findings for studies 1 through 4 in the main manuscript using repeated measures ANOVAs, which were part of our initial preregistered analysis for the studies. While we had deviated from our original plan (due to our data violating the assumption of sphericity crucial to ANOVAs) and instead used multilevel models for their greater appropriateness, we nevertheless conducted the original analyses prior to the shift. We report these findings for each study in which we had preregistered or conducted a repeated measures ANOVA analysis below. In doing so, we demonstrate that the results comparably support our hypothesis and convey our dedication to transparency. Finally, we also report the finding for our exploratory assessment of trait empathy moderating compassion ratings for AI and human-authored responses in study 1.

Experiment 1: Assessing the effect of vignette valence in compassion ratings (blind condition).

In experiment 1, where all participants were blinded to the response source, we had explored whether differences in third-party evaluations of compassion for select human and AI-generated responses were moderated by vignette valence. We ran a repeated measures ANOVA examining within-subjects compassion scores as a function of empathy source (AI v. Human response) and as a function of whether the responses from either source were in reaction to positive or negative vignettes.

The repeated measures analysis of variance showed a significant interaction, $F(1, 53) = 29.2$, $p < .001$, *partial* $\eta^2 = .36$, such that AI responses (M = 4.03, SE = .09) were rated as more compassionate compared to human responses (M = 3.71, SE = .08) for positive vignettes, $t(53) = 2.51$, $p = .015$. The same pattern was observed for responses to negative vignettes, with AI responses (M = 4.14, SE = .07) eliciting higher compassion ratings than human responses (M = 3.30, SE = .08), $t(53) = 7.69$, $p < .001$. These differences were larger for negative vignette responses, with a mean difference of 0.846, 95% CI = [4.00, 4.29] for AI and 95% CI = [3.13, 3.46] for humans.

Finally, when exploring the whether participants' compassion ratings for human and AI responses were moderated by their reported level of trait empathy, we found that trait empathy did not significantly moderate compassion ratings, $F(1, 52) = 1.92$, $p = 0.172$.

Experiment 2: Assessing differences in compassion ratings for AI and select human responses by transparency and valence.

In experiment 2, where participants were randomly assigned to evaluate select human and AI-authored responses under blind or transparent author label conditions, we had preregistered and ran a repeated measures ANOVA to assess differences in compassion ratings, with AI transparency as a between subjects condition and empathy source as a repeated measures condition. We further explored whether compassion ratings for each source differed as a function of valence by running a repeated measured ANOVA.

The repeated measures analysis of variance demonstrated a main effect for empathy source, $F(1, 195) = 63.2$, $p < .001$, *partial* $\eta^2 = .25$, such that the AI-generated responses (M = 4.06, SE = .04) were rated as more compassionate than the select human-generated responses (M = 3.60, SE = .04). We also found an interaction between source and transparency condition, $F(1, 195) = 10.8$, $p = .001$, $\eta^2 = .05$. Post hoc analyses indicated that within the blind condition, AI responses (M = 4.20, SE = .06) were rated as significantly more compassionate than human responses (M = 3.55, SE = .06), $t(195) = 7.93$, $p < .001$. When source identity was transparent, AI responses (M = 3.93, SE = .06) were still rated higher in compassion compared to human responses (M = 3.66, SE = .06), $t(195) = 3.30$, $p = .001$. The differences in compassion ratings were larger in the blind condition, with a mean difference of 0.649 (in contrast with a mean difference of 0.269 in the transparent condition), 95% CI = [4.08, 4.32] for AI and 95% CI = [3.44, 3.66] for humans.

In examining whether response valence moderated the interaction between author and condition, we did not find a significant 2x2x2 interaction, $F(1, 195) = .91$, $p = .341$, but we replicated the interaction between author and valence, $F(1, 195) = 71.9$ $p < .001$, *partial* $\eta^2 = .27$, such that for positive vignettes, AI responses (M = 3.99, SE = .05) received greater compassion scores compared to human responses (M = 3.74, SE = .04), $t(195) = 3.74$, $p < .001$. For negative vignettes, AI responses (M = 4.14, SE = .04) also had significantly higher compassion ratings than human responses (M = 3.47, SE = .04), $t(195) = 11.06$, $p < .001$. The differences in compassion ratings were larger for responses to negative vignettes, with a mean difference of 0.675 (v. 0.243 mean difference for responses to positive vignettes), 95% CI = [4.06, 4.22] for AI and 95% CI = [3.38, 3.55] for humans.

Experiment 3: Assessing differences in compassion ratings for AI and expert human responses by transparency and valence.

Experiment 3 had a design like experiment 2, but the human responses were created by trained hotline crisis responders, who we considered to be expert empathizers. We had preregistered and ran a repeated measures ANOVA to assess differences in compassion ratings, with AI transparency as a between subjects condition and empathy source as a repeated measures condition. We further explored whether compassion ratings for each source differed as a function of valence by running a repeated measured ANOVA.

Once again, we found a main effect of author, $F(1, 245) = 154.4$, $p < .001$, *partial* $\eta^2 = 0.39$ such that AI responses (M = 4.08, SE = .04) were rated as more compassionate than expert human responses (M = 3.47, SE = .03). As with Experiment 2, we also found a significant interaction between response source and the transparency condition, $F(1, 245) = 20.8$, $p < .001$, *partial* $\eta^2 = .08$. The between-subjects effect of transparency condition was significant as well, $F(1, 245) = 7.08$, $p = .008$, partial $\eta^2 = .03$, with both expert human and AI responses receiving higher compassion ratings in the blind condition (M = 3.84, SE = .03) than in the transparent condition (M = 3.71, SE = .04), though this effect was relatively small.

Post hoc tests indicated that when source identities weren’t shown (blind), AI responses (M = 4.26, SE = .05) were evaluated as more compassionate than expert human responses (M = 3.43, SE = .05), $t(245) = 12.13$, $p < .001$. When source identity was transparent, AI responses (M

= 3.90, SE = .05) still received higher compassion ratings compared to expert human responses (M = 3.52, SE = .05), t(245) = 5.50, $p < .001$. Again, the differences in compassion ratings were larger in the blind condition, with a mean difference of 0.832 (in contrast with a mean difference of 0.385 in the transparent condition), 95% CI = [4.16, 4.36] for AI and 95% CI = [3.34, 3.52] for humans.

In experiment 3, we again did not find a 2x2x2 interaction between response author, condition, and valence, F(1, 245) = .98, p = .324, but we replicated the author by valence interaction from study 2, F(1, 245) = 15.45, $p < .001$, *partial* η^2 = .06, such that AI responses (M = 4.05, SE = .04) were evaluated as more compassionate than expert human responses (M = 3.54, SE = .04), for both positive, t(245) = 9.49, $p < .001$, and negative scenarios (AI: M = 4.11, SE = .04 v. Human: M = 3.40, SE = .04), t(245) = 12.57, $p < .001$. Again, the differences in compassion ratings were larger for responses to negative vignettes, with a mean difference of 0.706 (v. 0.510 mean difference for responses to positive vignettes**)**, 95% CI = [4.04, 4.18] for AI and 95% CI = [3.32, 3.48] for humans.

Experiment 4: Assessing the effect of vignette valence in compassion and responsiveness ratings (transparent condition).

Experiment 4 was structured like experiment 1 and used a subset of the same human expert and AI responses as Experiment 3, but all the responses were transparently labeled. In addition, participants rated how responsive each response was in terms of relaying understanding, validation, and care. Regarding responsiveness, we hypothesized that AI responses would be rated as expressing greater responsiveness than empathic responses generated by expert humans (crisis line workers). Specifically, we hypothesized that AI responses would be rated as more understanding, validating, and caring. We preregistered a repeated measures ANOVA analysis to assess the effect of response valence on compassion and responsiveness evaluations for either response source.

We replicated the interaction effect between author and valence on compassion ratings with the ANOVA, F(1, 57) = 4.62, p = .036, *partial* η^2= .07, such that AI responses (M = 3.85, SE = .08) were evaluated as more compassionate than expert human responses (M = 3.50, SE = .08) for both positive, t(57) = 3.28, p = .002, and negative scenarios (AI: M = 3.97, SE = .07 v. Human: M = 3.33, SE = .09), t(57) = 5.77, $p < .001$. Again, the differences in compassion ratings were larger for responses to negative vignettes, with a mean difference of 0.644 (v. 0.356 mean difference for responses to positive vignettes**)**, 95% CI = [3.84, 4.10] for AI and 95% CI = [3.15, 3.51] for humans.

After further examining responsiveness, we found no significant author by valence interaction, F (1, 57) = 3.80, p = 0.056, *partial* η^2= .06. Despite the lack of significance, post hoc comparisons suggested that responsiveness ratings for AI responses were greater when they addressed negative circumstances, t(57) = 4.41, SE = .08, $p < .001$, than positive circumstances, t(57) = 2.94, SE = .06, p = .005, with a mean difference of 0.350 (v. 0.188 mean difference for responses to positive vignettes, 95% CI = [3.10, 3.36] for AI and 95% CI = [2.72, 3.04] for humans.

Supplementary References

1. Maisel, N. C., Gable, S. L. & Strachman, A. Responsive behaviors in good times and in bad. *Personal Relationships* **15**, 317–338 (2008).

2. *Participant Pool Management for universities. Sona Systems*. Sona Systems. http://www.sona-systems.com/ (2024).

3. Palan, S. & Schitter, C. Prolific.ac—A subject pool for online experiments. *Journal of Behavioral and Experimental Finance* **17**, 22–27 (2018).

4. Ayers, J. W. *et al.* Comparing Physician and Artificial Intelligence Chatbot Responses to Patient Questions Posted to a Public Social Media Forum. *JAMA Intern Med* **183**, 589 (2023).

5. Keaton, S. A. Interpersonal Reactivity Index (IRI): (Davis, 1980). in *The Sourcebook of Listening Research* (eds. Worthington, D. L. & Bodie, G. D.) 340–347 (Wiley, 2017). doi:10.1002/9781119102991.ch34.

6. Richard, F. D., Bond, C. F. & Stokes-Zoota, J. J. One Hundred Years of Social Psychology Quantitatively Described. *Review of General Psychology* **7**, 331–363 (2003).
