# Supplementary material for: Third-party evaluators perceive AI as more compassionate than expert humans
Source: Commun Psychol. 2025 Jan 10;3:4. doi: 10.1038/s44271-024-00182-6 (PMC11723910; doi:10.1038/s44271-024-00182-6)
Supplement: Supplementary file 3 — Reporting summary [file 44271_2024_182_MOESM3_ESM.pdf]

nature portfolio

Corresponding author(s): Michael Inzlicht

Last updated by author(s): Dec 7, 2024

Reporting Summary

Nature Portfolio wishes to improve the reproducibility of the work that we publish. This form provides structure for consistency and transparency in reporting. For further information on Nature Portfolio policies, see our Editorial Policies and the Editorial Policy Checklist.

Statistics

For all statistical analyses, confirm that the following items are present in the figure legend, table legend, main text, or Methods section.

n/a	Confirmed	
☐	☒	The exact sample size (n) for each experimental group/condition, given as a discrete number and unit of measurement
☐	☒	A statement on whether measurements were taken from distinct samples or whether the same sample was measured repeatedly
☐	☒	The statistical test(s) used AND whether they are one- or two-sided<br>*Only common tests should be described solely by name; describe more complex techniques in the Methods section.*
☐	☒	A description of all covariates tested
☐	☒	A description of any assumptions or corrections, such as tests of normality and adjustment for multiple comparisons
☐	☒	A full description of the statistical parameters including central tendency (e.g. means) or other basic estimates (e.g. regression coefficient) AND variation (e.g. standard deviation) or associated estimates of uncertainty (e.g. confidence intervals)
☐	☒	For null hypothesis testing, the test statistic (e.g. F, t, r) with confidence intervals, effect sizes, degrees of freedom and P value noted<br>*Give P values as exact values whenever suitable.*
☐	☒	For Bayesian analysis, information on the choice of priors and Markov chain Monte Carlo settings
☐	☒	For hierarchical and complex designs, identification of the appropriate level for tests and full reporting of outcomes
☐	☒	Estimates of effect sizes (e.g. Cohen's d, Pearson's r), indicating how they were calculated

Our web collection on statistics for biologists contains articles on many of the points above.

Software and code

Policy information about availability of computer code

Data collection	We programmed the studies using Qualtrics and collected data using the web platform Prolific Academic.
Data analysis	We analyzed the data using R 4.0.3.

For manuscripts utilizing custom algorithms or software that are central to the research but not yet described in published literature, software must be made available to editors and reviewers. We strongly encourage code deposition in a community repository (e.g. GitHub). See the Nature Portfolio guidelines for submitting code & software for further information.

Data

Policy information about availability of data

All manuscripts must include a data availability statement. This statement should provide the following information, where applicable:

- Accession codes, unique identifiers, or web links for publicly available datasets
- A description of any restrictions on data availability
- For clinical datasets or third party data, please ensure that the statement adheres to our policy

All data and code can be found on our Open Science Framework (OSF) repository: https://osf.io/wjx48/

Human research participants

Policy information about studies involving human research participants and Sex and Gender in Research.

Reporting on sex and gender	We did not consider sex and gender in the study design because we did not have a priori or preregistered analyses relating to sex or gender. However, sex information was collected based on self reporting (290 female, 260 male, 5 Prefer not to say/N/A) through Prolific Academic. This information is in the publicly available data, in the our supplementary table, and participants provided consent for sharing demographic data.
Population characteristics	Please refer to Table 1 in the main manuscript file for a detailed breakdown of the above demographic characteristics for participants in studies 1 through 4.
Recruitment	Participants were recruited through Prolific Academic and provided demographic information in accordance with the parameters set by Prolific Academic.
Ethics oversight	The study was approved by the Human Participant Ethics Protocol Submission at the University of Toronto.

Note that full information on the approval of the study protocol must also be provided in the manuscript.

Field-specific reporting

Please select the one below that is the best fit for your research. If you are not sure, read the appropriate sections before making your selection.

☐ Life sciences ☒ Behavioural & social sciences ☐ Ecological, evolutionary & environmental sciences

For a reference copy of the document with all sections, see nature.com/documents/nr-reporting-summary-flat.pdf

Behavioural & social sciences study design

All studies must disclose on these points even when the disclosure is negative.

Study description	The study used a repeated-measures design to compare AI-generated empathetic responses to human responses, evaluating third-party compassion, responsiveness, and preferences across four preregistered experiments under varying conditions of response author transparency.
Research sample	Our final sample consisted of 54 participants in study 1, 197 participants in study 2, 247 participants in study 3, and 58 participants in study 4. The participants were English-speaking and were recruited from Canadian and US samples on Prolific Academic.
Sampling strategy	In studies 1 and 4, which had a completely within-subject design, we aimed for a sample of 54 participants given that a power analysis suggests we'd achieve at least 80% power to detect the average effect size in social psychology of d = .4. For studies 2 and 3, where we had a mixed design with one between-subject and one within-subject variable, we aimed to run 400 participants, giving us 80% power to detect an interaction as small as f = 0.15 even after dropping inattentive participants.
Data collection	A total of 556 (54 participants in study 1, 197 in study 2, 247 in study 3, and 58 participants in study 4) Prolific participants accepted and completed the online experiments in their entirety. Participants did not know about the purpose and hypotheses of the study, but were debriefed upon their completion of the surveys.
Timing	Study 1 data was collected in September 2023, study 2 data in November 2023, study 3 data in February 2024, and study 4 data in May 2024; a separate set of participants were recruited for every study.
Data exclusions	We excluded participants who failed to pass one or both of the two attention checks that were at the beginning and end of the survey(s).
Non-participation	Any participants who expressed disagreement with their participation in the study, their understanding of the informed consent section of the survey, or did not agree with having their anonymized data made available on a research database were excluded. No data was provided by these participants thereafter.
Randomization	In experiments 2 and 3, participants were randomly assigned to either transparent or blind conditions in a between-subject design: in the transparent condition, they were told whether each response was generated by AI or humans; in the blind condition participants did not see the label for each response, so they could not immediately know which response was generated by a human or AI. Experiment 1 only had the blind condition, while study 4 only had the transparent condition.

Reporting for specific materials, systems and methods

We require information from authors about some types of materials, experimental systems and methods used in many studies. Here, indicate whether each material, system or method listed is relevant to your study. If you are not sure if a list item applies to your research, read the appropriate section before selecting a response.

Materials & experimental systems

n/a	Involved in the study
☒	☐ Antibodies
☒	☐ Eukaryotic cell lines
☒	☐ Palaeontology and archaeology
☒	☐ Animals and other organisms
☒	☐ Clinical data
☒	☐ Dual use research of concern

Methods

n/a	Involved in the study
☒	☐ ChIP-seq
☒	☐ Flow cytometry
☒	☐ MRI-based neuroimaging
